# Time trend analysis of HIV infection showing impact of national programs in an ICTC set up in a tertiary care hospital in North India

**DOI:** 10.1186/1471-2334-14-S3-P23

**Published:** 2014-05-27

**Authors:** Gurleen Metha, Amanpreet Kaur, Gurpreet Singh, Sunil K Arora

**Affiliations:** 1 Integrated Counseling and Testing Center (ICTC), Department of Immunopathology, PGIMER, Chandigarh, India

## Background

The analysis of changing trend in the HIV infection with respect to various factors in a tertiary care hospital of North India in an ICTC (Integrated Counseling and Testing Centre) setting was studied.

## Materials and methods

A total of 78,506 clients who visited the ICTC, PGIMER, Chandigarh from January 2003 to October 2013 were included and variables like age, sex, occupation, pattern of risk behavior and HIV sero status were studied. The patients were divided in three phases based on the phases of epidemic as evident from the variability in the positivity.

## Results

Variable trend is seen in the HIV infection in last 11 years. The change in positivity rate in terms of age has affected all the age groups. In terms of profession, during third phase there is a decrease in the businessman and truckers group probably due to very effective condom distribution program during NACPII. Least impact is found among students and laborers. The heterosexual route, IDU and blood, and blood products were stable while the homosexual route and parent to child (PPTCT) was still on the higher side.

## Conclusion

National intervention programs started during NACPII and continued during NACPIII have greatly impacted in stabilizing or even reducing the epidemic. However, more intensified epidemiological studies should be carried out which will help planning intervention projects to interrupt and control the transmission of HIV/ AIDS.

